# Maintenance Outcomes of the Children’s Healthy Living Program on Overweight, Obesity, and Acanthosis Nigricans Among Young Children in the US-Affiliated Pacific Region: A Randomized Clinical Trial

**DOI:** 10.1001/jamanetworkopen.2022.14802

**Published:** 2022-06-06

**Authors:** Rachel Novotny, Ashley B. Yamanaka, Jean Butel, Carol J. Boushey, Rica Dela Cruz, Tanisha Aflague, Patricia Coleman, Leslie Shallcross, Travis Fleming, Lynne R. Wilkens

**Affiliations:** 1 Department of Human Nutrition, Food and Animal Sciences, College of Tropical Agriculture and Human Resources, University of Hawai‘i at Mānoa, Honolulu; 2 Population Sciences in the Pacific Program, University of Hawai‘i Cancer Center, Honolulu; 3 Cooperative Extension and Outreach, College of Natural & Applied Sciences, University of Guam, Mangilao; 4 Cooperative Research, Extension, and Education Services, Northern Marianas College, Saipan; 5 Health, Home and Family Development, Institute of Agriculture, Natural Resources and Extension, University of Alaska, Fairbanks; 6 Community and Natural Resources Division (Land Grant Program), American Samoa Community College, Pago Pago

## Abstract

**Importance:**

Few obesity prevention trials among children have demonstrated sustainable outcomes in the long term.

**Objectives:**

To sustain a community-wide decrease in the prevalence of overweight and obesity among young children in the US-affiliated Pacific region.

**Design, Setting, and Participants:**

In the Children’s Healthy Living community-randomized clinical trial, hierarchical modeling comparing the change in intervention and control communities accounted for community randomization (community clustering with jurisdictions), and adjusted for the age and sex distribution of the assessed children in a cross-sectional design. The outcome measures were repeated in communities rather than among individual children. A total of 27 communities in 5 jurisdictions (Hawai‘i, Alaska, Commonwealth of the Northern Mariana Islands, American Samoa, and Guam) of the US-affiliated Pacific region were included. Participants included children aged 2 to 8 years in the 27 selected communities from October 1, 2012 (4329 in time 1 [baseline]) to August 31, 2015 (4043 in time 2 [intervention end]) and from January 1, 2019, to April 30, 2020 (1469 in time 3 [maintenance period]). Study analysis was completed March 25, 2022.

**Interventions:**

Nineteen activities addressed training, policies, systems, and environments of communities and 6 target behaviors of children (consumption of fruit and vegetables, water, and sugar-sweetened beverages; sleep; physical activity; and screen time) during a 2-year intervention period. Continued partnership with community coalitions, ongoing academic training of community partners, and use of trial data during a 6-year maintenance period.

**Main Outcomes and Measures:**

The primary outcome was measured anthropometry; secondary outcomes were the presence of acanthosis nigricans, dietary intake derived from 2 days of food records, and survey questions on screen time and sleep disturbance.

**Results:**

Among the 9840 children included in the analysis (4866 girls [49.5%] and 4974 boys [50.5%]; 6334 [64.4%] aged 2-5 years), the intervention group showed significant improvements compared with the control group from times 1 to 3 in prevalence of overweight plus obesity (*d* = −12.60% [95% CI, −20.92% to −4.28%]), waist circumference (*d* = −1.64 [95% CI, −2.87 to −0.41] cm), and acanthosis nigricans prevalence (*d* = −3.55% [95% CI, −6.17% to −0.92%]). Significant improvements were also observed from times 2 to 3 in prevalence of overweight plus obesity (*d* = −8.73% [95% CI, −15.86% to −1.60%]) but not in waist circumference (*d* = −0.81 [95% CI, −1.85 to 0.23] cm).

**Conclusions and Relevance:**

This randomized clinical trial found that the outcomes of the Children’s Healthy Living intervention were maintained and enhanced 6 years after the intervention among young children in the US-affiliated Pacific region. The prevalence of overweight, obesity, and acanthosis nigricans was further reduced in communities, suggesting that multilevel multicomponent interventions may help reduce child overweight and obesity in this region.

**Trial Registration:**

ClinicalTrials.gov Identifier: NCT01881373

## Key Points

**Question:**

Are the outcomes of the Children’s Healthy Living (CHL) trial on overweight and obesity among young children in the US-affiliated Pacific region maintained after 6 years?

**Findings:**

The CHL randomized clinical trial of 9840 participants found that the prevalence of overweight and obesity decreased significantly by 12.60% 6 years after baseline and by 8.73% 4 years after the end of the 2-year intervention among children aged 2 to 8 years.

**Meaning:**

These findings suggest that a multilevel, multicomponent approach helps reduce the prevalence of overweight and obesity among young children in the long term.

## Teaser

The Children’s Healthy Living community-randomized clinical trial assesses whether the trial intervention continues to reduce the prevalence of overweight and obesity among young children in the US-affiliated Pacific region after 6 years.

## Introduction

The prevalence of obesity and type 2 diabetes among adults in the Pacific region is among the highest in the world, leading to calls for action.^1^ The Children’s Healthy Living (CHL) Program focuses on prevention of childhood obesity in the Pacific region, which is known to track into obesity and type 2 diabetes in adulthood.^2^ In 2013, obesity in the US-affiliated Pacific (USAP) jurisdictions was 14% and was higher among children aged 6 to 8 years than those aged 2 to 5 years^3^; in addition, obesity is associated with occurrence of acanthosis nigricans, an indicator of insulin resistance.^4,5^ These findings indicate the metabolic significance of obesity, even in childhood.

Interventions to change behavior alone have not been sufficient to reduce the prevalence of obesity or type 2 diabetes; rather, broad changes in the obesogenic environment are needed to affect population levels of obesity.^6^ Four trials before the CHL Program made such changes.^7,8,9,10^ Three of these trials—the Bright Start,^7^ Shape Up Somerville,^8^ and Romp & Chomp^9^ interventions—targeted eating and physical activity through multilevel interventions addressing the school environment and/or policy and measured the effects on child overweight or obesity, similar to the CHL Program. Bright Start^7^ showed a 10% decrease in obesity prevalence; Shape Up Somerville,^8^ a 0.10 decrease in body mass index (BMI; calculated as weight in kilograms divided by height in meters squared) *z* scores among children aged 3 years; and Romp & Chomp^9^ showed lower mean weight, BMI, and BMI *z* scores in the subsample of children aged 3.5 years and lower prevalence of overweight and obesity in the subsamples aged 2 and 3.5 years compared with the control group. Later, the Dietary- and Lifestyle-Induced Health Effects in Children and Infants multilevel study^10^ was conducted in multiple communities in 8 European countries. This intervention did not achieve a significant change in BMI *z* scores, waist-to-height ratio, or body fat percentage,^11^ and no overall significant behavioral changes were observed.^10^

The CHL trial used a social ecological framework of health and wellness designed to act on multiple levels and in multiple components within behavioral, physical, social, cultural, economic, and policy environments^12^ to reduce the prevalence of overweight and obesity among children in multiple USAP communities, ultimately reducing it by 3.9% during a 2-year period.^13^ The trial was guided by the ANGELO (Analysis Grid for Elements Linked to Obesity) framework^14^ and designed by merging community input and evidence-based strategies.^12,15,16^ It sought to identify and build on what was currently working in communities by engaging community partners and members in ways sensitive to their culture and putting the health and well-being of young children at the forefront of community decisions and actions in a way that could be sustained through the land grant college network and local coalitions.^17,18^ In addition, the trial developed and used a template for implementation and had exceptional accuracy of anthropometric measurements.^19^ It was also novel in terms of the diversity and range of understudied racial and ethnic groups (ie, Alaska Native, Native Hawaiian, and Pacific Islander) and regions (ie, USAP jurisdictions) included in a single trial with populations at high risk for chronic disease.^20^

Few obesity prevention studies have included long-term follow-up, described intervention maintenance, and/or reported follow-up analyses after completion of the study intervention program.^21,22,23,24^ More evidence is needed to understand obesity prevention intervention sustainability and its long-term effects. We hypothesized that the CHL trial would maintain a reduction in the prevalence of overweight and obesity among young children in the USAP region.

## Methods

### Study Population

**Figure 1.**
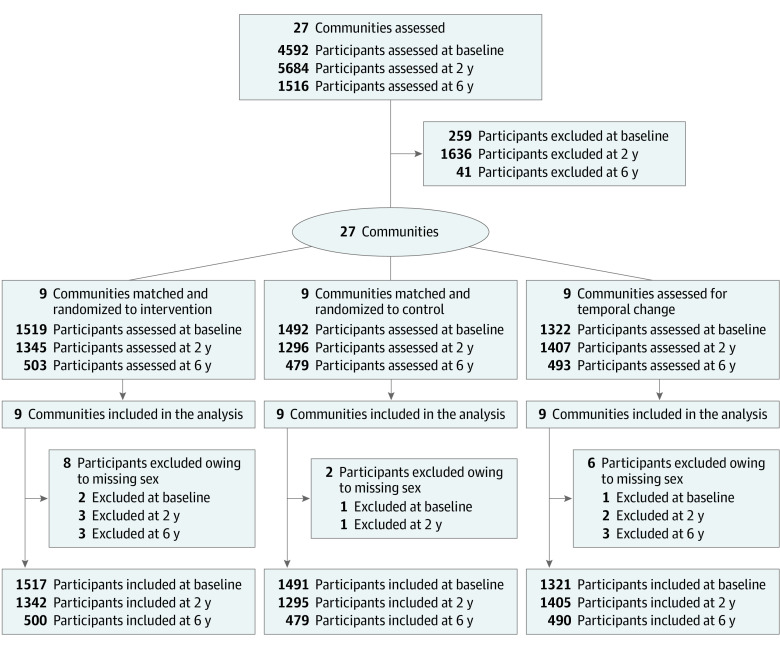
CONSORT Diagram for the Children’s Healthy Living Trial

In 2019-2020, the 27 communities in 5 USAP jurisdictions (American Samoa, Alaska, Hawai‘i, Commonwealth of the Northern Mariana Islands, and Guam) originally included in the CHL trial underwent reassessment. Initial study community selection criteria based on the 2000 US census were more than 15% of the population consisting of individuals of Indigenous or Native descent (ie, native to each jurisdiction), more than 10% of the population younger than 10 years, population size greater than 1000, and reasonable geographical accessibility.^13^ Eighteen selected communities were matched for size and randomized within each jurisdiction to an intervention or a control (delayed optimized intervention) group. One or 2 communities per jurisdiction that met the eligibility criteria but were less amenable to matching were retained as temporal communities, with only demographic characteristics and anthropometry measured to observe trends over time. Matched communities were randomly assigned to the intervention or the control by a computer program and delivered in envelopes to the jurisdictions/communities. The trial protocol is provided in Supplement 1. The study adhered to Consolidated Standards of Reporting Trials (CONSORT) reporting guideline; a study flowchart is presented in Figure 1.^21^ Institutional review board approval was obtained from the University of Hawai‘i at Mānoa, University of Guam, and the University of Alaska at Fairbanks. Northern Marianas College and American Samoa Community College ceded approval to the University of Hawai‘i. Informed consent was obtained from caregivers and assent from the child participants.

A total of 4333 children aged 2 to 8 years were recruited for measurements at schools or community events at time 1; 4048, at time 2; and 1475, at time 3 (Figure 1). The recruitment goal for times 1 and 2 of 150 children per community for anthropometry and 100 for dietary and physical activity assessment was established to detect effect sizes of 0.26 to 0.31, assuming α = .05 (2-sided), β = 0.20, and an intraclass correlation coefficient within communities of 0.02.^18,21^ The goal of 50 per community for time 3 was set to detect differences compared with times 1 or 2 with effect sizes of 0.15 and 0.18, assuming the observed intraclass correlation coefficient of 0.005.

The samples at times 1, 2, and 3 were independent samples of children from the communities (not repeated measures). The mean interval from times 1 to 2 measurements was 26.0 (range across communities, 21.2-28.6) months; from times 1 to 3 measurements, 78.5 (range across communities, 70.7-82.8) months. The numbers included in analysis, after exclusion of children outside the age range of 2 to 8 years, were 4329 for time 1, 4042 for time 2, and 1469 for time 3 (Figure 1). Table 1 shows the sample characteristics by intervention period. eTable 1 in Supplement 2 shows the sample sizes by measurement module collected.

**Table 1.** Child Characteristics in the Children’s Healthy Living Program by Study Time Period and Community Group in the US-Affiliated Pacific Region, 2012-2020

Characteristic	Community group, No. (%) of children^a^									
	Intervention			Control			Temporal			All (N = 9840)
	Time 1 (n = 1517)	Time 2 (n = 1342)	Time 3 (n = 500)	Time 1 (n = 1491)	Time 2 (n = 1295)	Time 3 (n = 479)	Time 1 (n = 1321)	Time 2 (n = 1405)	Time 3 (n = 490)	
Age group, y										
2-5	952 (62.7)	825 (61.5)	325 (65.0)	930 (62.4)	735 (56.7)	303 (63.3)	996 (75.4)	930 (66.2)	338 (69.0)	6334 (64.4)
6-8	565 (37.2)	517 (38.5)	175 (35.0)	561 (37.6)	560 (43.2)	176 (36.7)	325 (24.6)	475 (33.8)	152 (31.0)	3506 (35.6)
Sex										
Boys	757 (49.9)	674 (50.2)	229 (45.8)	771 (51.7)	660 (51.0)	238 (49.7)	701 (53.1)	701 (49.9)	243 (49.6)	4974 (50.6)
Girls	760 (50.1)	668 (49.8)	271 (54.2)	720 (48.3)	635 (49.0)	241 (50.3)	620 (46.9)	704 (50.1)	247 (50.4)	4866 (49.5)
Hispanic ethnicity										
Yes	173 (11.4)	92 (6.9)	65 (13.0)	136 (9.1)	123 (9.5)	45 (9.4)	120 (9.1)	129 (9.2)	63 (12.9)	946 (9.6)
No	1340 (88.3)	1250 (93.1)	435 (87.0)	1355 (90.9)	1172 (90.5)	434 (90.6)	1197 (90.6)	1276 (90.8)	427 (87.1)	8886 (90.3)
Unknown	4 (0.3)	0	0	0	0	0	4 (0.3)	0	0	8 (0.1)
Race and other ethnicity										
American Indian or Alaska Native	24 (1.6)	9 (0.7)	2 (0.4)	19 (1.3)	8 (0.6)	1 (0.2)	81 (6.1)	79 (5.6)	55 (11.2)	278 (2.8)
Asian	85 (5.6)	74 (5.5)	27 (5.4)	195 (13.1)	159 (12.3)	56 (11.7)	197 (14.9)	204 (14.5)	67 (13.7)	1064 (10.8)
Black	6 (0.4)	17 (1.3)	9 (1.8)	1 (0.1)	7 (0.5)	0	8 (0.6)	9 (0.6)	0	57 (0.6)
Native Hawaiian or Pacific Islander	898 (59.2)	774 (57.7)	268 (53.6)	809 (54.3)	687 (53.1)	239 (49.9)	599 (45.3)	506 (36.0)	176 (35.9)	4956 (50.4)
White	120 (7.9)	138 (10.3)	44 (8.8)	147 (9.9)	139 (10.7)	42 (8.8)	151 (11.4)	182 (13.0)	30 (6.1)	993 (10.1)
>1 race	374 (24.7)	318 (23.7)	150 (30.0)	317 (21.3)	283 (21.9)	135 (28.2)	282 (21.3)	409 (29.1)	157 (32.0)	2425 (24.6)
Unknown	10 (0.7)	12 (0.9)	0	3 (0.2)	12 (0.9)	6 (1.3)	3 (0.2)	16 (1.1)	5 (1.0)	67 (0.7)
Indigenous^b^										
Yes	1087 (71.7)	871 (64.9)	320 (64.0)	968 (64.9)	759 (58.6)	306 (63.9)	793 (60.0)	852 (60.6)	343 (70.0)	6299 (64.0)
No	430 (28.3)	471 (35.1)	180 (36.0)	523 (35.1)	536 (41.4)	173 (36.1)	528 (40.0)	553 (39.4)	147 (30.0)	3541 (36.0)

^a^ Percentages have been rounded and may not total 100.

^b^ Defined as native race or ethnicity of jurisdiction.

### The CHL Intervention

The CHL intervention was developed by a consortium of collaborators at land grant colleges in the USAP region.^22^ The CHL trial used a common template of 19 activities that were selected to address target behaviors; these activities were derived from community-informed ideas^17^ and blended with approaches from successful interventions from the literature.^12^ Implementation focused on supporting existing community programs to expand or innovate based on the template using positive deviance strategies.^23,24^ Each intervention community had approximately $50 000 to purchase supplies and materials and at least 1 staff member to guide community intervention activities. Each jurisdiction employed 1 staff member to coordinate the intervention, and the coordinating center consisted of 2 part-time leads and 1 intervention coordinator. The intervention activities were grouped into 4 functions (or strategies): environmental change, organizational policy change, social marketing, and training (Table 2) that addressed the interpersonal (training role models, parents, and teachers), community (increasing access to healthy foods and environments for safe play), and organizational and policy (strengthening preschool wellness policies) levels of the social ecological model. The intervention spanned a 2-year period (January 1, 2013, through December 31, 2014), with monthly process measures collected and used to calculate dose and fidelity^24^ in each of the 9 intervention communities (Table 2). These measures came from monthly reports submitted to the CHL coordinating center based on RE-AIM components,^25^ which categorized activities based on the 4 strategies of policy, environment, capacity building, and promotion.

**Table 2.** The CHL Intervention Strategies and Activities Sustained at 6-Month Follow-up, in Communities, by Jurisdiction^a^

Strategy and activities	Jurisdiction, No. of communities					No. (%) of all communities
	Alaska	American Samoa	Commonwealth of the Northern Mariana Islands	Guam	Hawai‘i	
**Review assessment data on policy and the physical environment related to the 6 CHL behaviors**						
Review data on existence and quality of preschool wellness policy:						
Review preschool wellness policy assessment data to identify policy gaps	0	0	0	0	0	0
Address policy gaps with preschool administration	0	0	0	0	0	0
Assess policy implementation quality (identify strengths and weaknesses)	0	0	0	0	0	0
Work with preschool administrators to address weaknesses in policy implementation	0	0	2	0	2	4 (44.4)
Review baseline data on physical environment from CAT						
Assess the physical environment using the CAT	0	0	0	0	0	0
Review CAT data related to the physical environment to identify areas for improvements and advocacy	0	0	0	0	0	0
Improve CAT indicated physical activity environments	1	0	0	2	2	5 (55.5)
Advocate (with partners, stakeholders, role models, coalitions) for CAT-indicated physical activity environment changes	0	0	2	0	0	2 (22.2)
**Partner and advocate for environmental change**						
Work with organizations/coalitions to advocate for:						
Better access to parks that are safe and inviting	1	0	2	2	0	5 (55.5)
Better access to clean water	0	0	0	0	1	1 (11.1)
Safer environments for walking, biking, etc (eg, bike lanes and racks, sidewalks, greenways)	1	0	0	2	2	5 (55.5)
Better food placement/availability	0	0	0	0	2	2 (22.2)
Gardens and hydroponics	0	2	0	2	2	6 (66.7)
Partner with existing entities to purchase or obtain sponsorship for:						
Water in the preschools and childcare centers	0	0	0	0	0	0
Gardening supplies for preschool kids	0	0	0	2	0	2 (22.2)
Sports equipment for preschool kids	1	0	0	2	0	3 (33.3)
Campaigns and messages	0	0	0	2	0	2 (22.2)
**Promote the CHL message**						
Support role models to deliver CHL messages in various ways (using the CHL role model curriculum as a guide)	0	2	2	2	0	6 (66.7)
Enhance exiting social marketing campaigns in the intervention communities, and/or develop low-cost local social marking campaigns related to the 6 CHL behaviors	0	0	0	2	0	2 (22.2)
Advertise CHL or other activities that promote 6 CHL target behaviors	1	2	1	2	1	7 (77.8)
**Train the trainers**						
Train individuals to promote gardening in preschools and communities	0	0	0	2	2	4 (44.4)
Train individuals to lead interactive, hands-on sessions to promote the 6 CHL behaviors	0	0	0	2	2	4 (44.4)
Train individuals to organize and lead family-based activities that support the 6 CHL behaviors (park clean-ups, hikes, cooking sessions)	0	0	0	2	1	3 (33.3)
Provide training to preschool and childcare staff on wellness policies	0	0	2	2	2	6 (66.7)
Train childcare providers and preschool teachers in curricula related to 6 CHL behaviors	0	0	0	2	1	3 (33.3)
Train role models (community champions, role celebrities, role models)	0	0	2	2	1	5 (55.5)

Abbreviations: CAT, Community Assessment Toolbox; CHL, Children’s Healthy Living.

^a^ Alaska had 1 community; all other jurisdictions had 2 communities.

The CHL Program implemented activities that worked (based on fidelity and qualitative data) for the intervention communities in the control (delayed optimized intervention) communities for the 6 months after the 2-year intervention phase of the program was completed. Using the RE-AIM framework,^26^ implementation fidelity data, and qualitative data, the activities were ranked as follows: (1) community acceptability of the activity; (2) the reach the activity had on the target audience; (3) likelihood of effectiveness of the activity; (4) adoption of the activity by community partners; (5) sustainability of the activity in the community; and (6) feasibility of implementing the activity in the 6-month time frame. The optimized community plan included 8 activities with at least 1 activity from each CHL strategy and addressed multiple social and environmental levels.

### Intervention Maintenance

Maintenance activities involved continued partnership with community coalitions, support of ongoing academic training, use of data gathered during the study for research publications, and program and policy planning. A CHL Center of Excellence renewal grant collected time 3 data and sustained annual meetings, monthly Program Steering Committee meetings, access to CHL data, presentation of key data on the website, and maintenance of the CHL website as a portal to CHL resources. A land grant college CHL Network (CHLN) multistate project further facilitated ongoing collaboration and evolution of the CHL Program.^27^

Maintenance of CHL intervention activities in intervention communities was assessed at 6 months post intervention. Each of the 4 intervention strategies was maintained with some activity. Capacity building through train-the-trainer approaches and support for role models to deliver the CHL message were maintained in at least 6 of the 9 intervention communities. Also, 7 of the 9 intervention communities continued to advertise CHL-related activities. After the CHL intervention period (time 2) was completed, CHL continued to develop capacity in the region through childhood obesity courses offered through the CHL Summer Institute, anthropometric standardization trainings, and disseminating CHL data and intervention materials through the CHL website. Dissemination of CHL intervention included providing community partners with community reports of CHL data, data visualizations of CHL data on the CHL website, and incorporation of CHL intervention into the SNAP-Ed Toolkit.^28^ Capacity building continued through training grants, CHL Summer Institute courses, anthropometric trainings, and CHL intervention train-the-trainer activities with community partners. The CHLN project began in 2016 with members from the CHL intervention jurisdictions, with the addition of newer members extending reach. The CHLN continued the connection between the land grant colleges and promoted the health and well-being of young children in the CHL jurisdictions and beyond; collaboration with community and jurisdiction partners was continued by CHLN members who had been part of the CHL intervention. Examples of collaborations were physical activity training with schools, promoting policies through active participation in obesity task forces or noncommunicable disease groups, working with other research projects, and incorporating CHL intervention activities in the jurisdiction’s federal nutrition programs.

After the 6-month postintervention survey, CHL intervention activity tracking was accomplished with CHLN annual reports.^27^ The CHLN reports, however, do not break down which of these activities were conducted in intervention communities and which activities were conducted throughout the jurisdiction or region.

### Outcomes

The primary analysis of the CHL intervention compared changes from times 1 to 2 in outcomes of child body size, acanthosis nigricans, and behaviors including food intake, sleep, physical activity, and screen time among intervention, control, and temporal communities.^13^ This prespecified maintenance analysis repeated this analysis comparing time 1 or time 2 with time 3 for all outcomes except physical activity, because accelerometry was not included at time 3 data collection, and no intervention effect size (time 2 minus time 1) had been found for the accelerometry outcomes of metabolic equivalent task hours or moderate to vigorous activity per day. Key CHL measurement tools and outcome measures were described previously in detail^19,21^ and are described briefly in the following sections.

#### Primary Outcomes

Body size measures included weight, height, and waist circumference and the calculations of BMI, BMI *z* score, and BMI percentile relative to Centers for Disease Control and Prevention reference data.^29,30^ Biologically implausible *z* score values of less than −8 SD and greater than 4 SD were excluded from analysis for BMI and waist circumference, based on guidance from the National Health and Nutrition Examination Survey reference values.^29,30^ Overweight was defined as BMI in the 85th to 94th percentile and obesity was defined as BMI greater than or equal to the 95th percentile for age and sex. Children with healthy weight (BMI, 5th-84th percentile) were the reference group in the analysis of overweight and obesity, and children with underweight (BMI <5th percentile for age and sex) were excluded. Two hundred sixty-one of 9840 children (2.6%) were underweight, with 92 of 4329 (2.1%) at time 1, 119 of 4042 (2.9%) at time 2, and 50 of 1469 (3.4%) at time 3. Staff in all jurisdictions used standard instruments from times 1 and 2^19^ and were again required to display good agreement compared with an expert in anthropometry training.^31,32^

#### Secondary Outcomes

Acanthosis nigricans is a skin indicator of insulin resistance^5^ that was assessed at the back of the neck by trained staff using the scale developed by Burke et al.^33^ Sleep quality was measured with the Tayside Sleep Disturbance Questionnaire,^34^ and sleep duration was reported by the caregiver as hours per day asleep at night plus naps. Sleep durations less than 5 h/d were excluded from analysis. Screen time, defined as hours spent interacting with screens, such as television and computer games, was reported by the parents.

Food groups of the children’s diet were calculated from 2 dietary records on randomly selected days to ensure representation of all days of the week across children. The records were completed by the parent or caregiver, with assistance from other child caregivers as described previously.^35^ These data were entered into the Pacific Tracker, version 3, a dietary and physical activity assessment system,^36^ which includes the food composition database maintained by the Nutrition Support Shared Resource at the University of Hawai‘i Cancer Center, which in turn includes information on local foods in the Pacific region.^37^ The mean dietary components were calculated across days, weighted for weekdays and weekend days, and adjusted for within-person variance.^31^

Caregiver respondents for the children completed questionnaires about other demographics, including race and ethnicity categorized according to the US Office of Management and Budget (OMB) guidelines. An Indigenous variable was defined as reporting the Indigenous ethnic group of the jurisdiction where the data were being collected (for Alaska, Alaska Native [Yupik and Inupiaq]; for Commonwealth of the Northern Marianas, Chamorro and Carolinian; for Guam, Chamorro; for Hawai‘i, Native Hawaiian; and for American Samoa, Samoan), either alone or with other races and ethnicities.

### Statistical Analysis

Data analysis was completed March 25, 2022. Hierarchical difference-in-difference marginal models were used to estimate means at each time point by randomization group. The models accounted for the study design, using community as the unit for hypothesis testing and accounting for the community clusters within jurisdiction strata. The models were adjusted for sex and age (in months) and were weighted to account for the number of children in the community. Race was not adjusted for, because it was highly colinear with jurisdiction. Models of dichotomous outcomes of overweight and obesity prevalence and acanthosis nigricans used a logistic link, whereas models of continuous outcomes of BMI *z* score, waist circumference, and each target behavior (consumption of fruit, vegetables, sugar-sweetened beverages, and drinking water; screen time; sleep time; and sleep disturbance) used a linear link. Assumptions of the linear statistical models were checked and, if not met, Box-Cox transformations were applied for the outcome. If results were similar, untransformed models were displayed. Transformations were used in final models for waist circumference, water intake, sleep disturbance, and screen time. Changes by randomization group over time and the differences in changes between groups were assessed by a Wald test with degrees of freedom based on the number of communities. Three comparisons are presented: the primary intervention effect size from times 1 to 2 is provided for context; the sustained intervention effect size, from times 1 and 3; and the maintenance effect size, from times 2 to 3. The results given for the primary intervention effect size (times 1 vs 2) are slightly different than previously published results^13^ because the sex and age adjustment parameters were modified by inclusion of time 3 in the model. Two-sided *P* < .05 was considered statistically significant. Data analysis was performed using SAS, version 9.4 (SAS Institute, Inc).

## Results

### Demographics

For the 9840 participants throughout the CHL, 6334 children (64.4%) were in the group aged 2 to 5 years, and 3506 (35.6%) were in the group aged 6 to 8 years (Table 1); 4866 (49.5%) were girls and 4974 (50.5%) were boys. A total of 6299 children (64.0%) were classified as Indigenous to their jurisdiction; this percentage was higher than the 4956 (50.4%) classified as Native Hawaiian and Pacific Islander by the OMB classification, because many individuals identified as Indigenous were classified as being of more than 1 race or ethnicity by the OMB. In addition, 278 children (2.8%) were identified as American Indian or Alaska Native, 1064 (10.8%) as Asian, 57 (0.6%) as Black, 993 (10.1%) as White, 2425 (24.6%) as more than 1 race, and 67 (0.7%) as unknown race or ethnicity; 946 (9.6%) reported Hispanic ethnicity.

### Primary Outcomes

**Figure 2.**
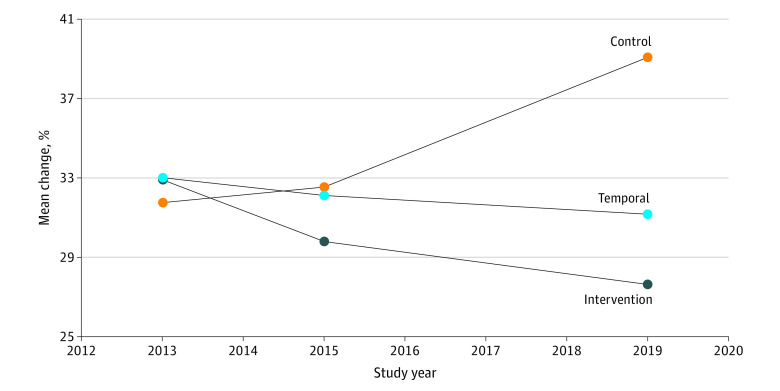
Prevalence of Overweight and Obesity Among Intervention, Control, and Temporal Communities in the Children’s Healthy Living Trial in the US-Affiliated Pacific Region, 2012-2020

Obesity-related outcomes of the CHL intervention communities are provided in Table 3, and change in overweight and obesity prevalence across time is presented in Figure 2. The CHL trial yielded significant differences between the intervention and control communities for prevalence of overweight and obesity from times 1 to 3 of −12.60% (95% CI, −20.92% to −4.28%; *P* = .003), for times 1 to 2, −3.88% (95% CI, −5.31% to −0.87%; *P* = .03), and for times 2 to 3, −8.73% (95% CI, −15.86% to −1.60%; *P* = .02). Differences in waist circumference between the intervention and control communities were significant from times 1 to 3 at −1.64 cm (95% CI, −2.87 to −0.41 cm; *P* = .01) but not from times 1 to 2 (−0.83 cm [95% CI, −1.51 to −0.15 cm; *P* = .02]) or times 2 to 3 (−0.81 cm [95% CI, −1.85 to 0.23 cm; *P* = .13]). Mean BMI *z* score changed by −0.25 SD units (95% CI, −0.43 to −0.08; *P* = .007) from times 1 to 3, with change from times 1 to 2 of −0.04 SD units (95% CI, −0.15 to 0.06; *P* = .39) and from times 2 to 3 of −0.21 SD units (95% CI, −0.34 to −0.07; *P* = .004).

**Table 3.** The Children’s Healthy Living Trial Changes in Measures Among Communities by Time Points in US-Affiliated Pacific Region, 2012-2020^a^

Child measure	No. of children included	Community														
		Intervention			Control			Temporal			Intervention vs control					
		T1	T2	T3	T1	T2	T3	T1	T2	T3	Difference, T2 vs T1	*P* value	Difference, T3 vs T1	*P* value	Difference, T3 vs T2	*P* value
Anthropometry																
Overweight and obesity prevalence, %	9473	32.91 (29.13 to 36.94)	29.80 (26.60 to 33.26)	27.63 (23.25 to 32.48)	31.76 (27.23 to 36.67)	32.54 (28.98 to 36.33)	39.08 (34.07 to 44.33)	33.01 (26.68 to 40.04)	32.12 (24.07 to 41.40)	31.17 (22.58 to 41.28)	−3.88 (−7.29 to −0.47)	.02	−12.60 (−20.92 to 4.28)	.003	−8.73 (−15.86 to 1.60)	.02
BMI *z* score	9473	0.58 (0.44 to 0.72)	0.50 (0.37 to 0.63)	0.46 (0.30 to 0.62)	0.55 (0.39 to 0.72)	0.51 (0.36 to 0.67)	0.68 (0.53 to 0.83)	0.59 (0.38 to 0.80)	0.52 (0.25 to 0.78)	0.50 (0.19 to 0.80)	−0.04 (−0.15 to 0.06)	.39	−0.25 (−0.43 to −0.08)	.007	−0.21 (−0.34 to −0.07)	.003
Waist circumference, cm^b^	9308	55.04 (54.22 to 55.90)	54.90 (53.87 to 56.00)	55.86 (54.87 to 56.92)	54.39 (53.43 to 55.41)	55.08 (54.01 to 56.23)	56.86 (55.84 to 57.92)	54.83 (53.44 to 56.34)	55.46 (53.59 to 57.54)	56.03 (54.21 to 58.04)	−0.83 (−1.51 to −0.15)	.02	−1.64 (−2.87 to −0.41)	.009	−0.81 (−1.85 to 0.23)	.13
Acanthosis nigricans prevalence, %	6544	5.55 (2.69 to 11.09)	2.00 (0.67 to 5.88)	1.52 (0.87 to 2.65)	3.45 (1.80 to 6.50)	6.03 (4.28 to 8.43)	2.96 (1.61 to 5.40)	NM	NM	NM	−6.12 (−10.56 to −1.68)	.007	−3.55 (−6.17 to −0.92)	.008	2.57 (−3.61 to 8.76)	.42
Behaviors																
Sleep, h/d^b^	9724	8.75 (8.15 to 9.34)	9.19 (8.78 to 9.59)	8.94 (8.62 to 9.26)	9.04 (8.60 to 9.47)	9.60 (9.25 to 9.95)	9.03 (8.55 to 9.52)	9.06 (8.47 to 9.65)	9.46 (8.86 to 10.06)	9.08 (8.58 to 9.57)	−0.12 (−1.16 to 0.91)	.80	0.20 (−0.25 to 0.65)	.37	0.32 (−0.57 to 1.22)	.46
Sleep disturbance score^b^^,^^c^	6461	5.03 (4.56 to 5.53)	4.67 (4.15 to 5.24)	6.86 (6.23 to 7.54)	5.33 (4.77 to 5.93)	5.65 (4.99 to 6.37)	6.79 (5.07 to 8.74)	NM	NM	NM	−0.68 (−1.40 to 0.04)	.06	0.37 (−1.32 to 2.15)	.64	1.05 (−0.22 to 2.42)	.10
Screen time, h/d^b^	6461	3.97 (3.47 to 4.51)	3.81 (3.34 to 4.31)	3.98 (3.38 to 4.63)	4.01 (3.43 to 4.65)	4.32 (3.70 to 5.01)	4.11 (3.72 to 4.53)	NM	NM	NM	−0.48 (−1.06 to 0.11)	.11	−0.09 (−0.88 to 0.69)	.81	0.38 (−0.42 to 1.18)	.35
Dietary consumption, cups/d																
Fruit	4966	1.03 (0.90 to 1.15)	0.95 (0.85 to 1.05)	1.05 (0.84 to 1.25)	1.09 (0.98 to 1.19)	1.04 (0.91 to 1.17)	0.90 (0.66 to 1.15)	NM	NM	NM	−0.03 (−0.13 to 0.07)	.54	0.21 (−0.06 to 0.47)	.11	0.24 (−0.03 to 0.50)	.07
Vegetables	4966	0.69 (0.62 to 0.76)	0.68 (0.63 to 0.74)	0.69 (0.62 to 0.76)	0.71 (0.63 to 0.79)	0.71 (0.65 to 0.76)	0.65 (0.54 to 0.76)	NM	NM	NM	0 (−0.08 to 0.07)	.89	0.06 (−0.09 to 0.21)	.40	0.06 (−0.07 to 0.20)	.32
Water^b^	4966	1.23 (1.21 to 1.26)	1.24 (1.22 to 1.26)	1.25 (1.20 to 1.30)	1.24 (1.22 to 1.26)	1.25 (1.23 to 1.27)	1.24 (1.20 to 1.29)	NM	NM	NM	0 (−0.03 to 0.02)	.70	0.02 (−0.07 to 0.11)	.69	0.02 (−0.05 to 0.10)	.56
SSBs	4966	0.66 (0.53 to 0.79)	0.61 (0.49 to 0.73)	0.61 (0.38 to 0.85)	0.66 (0.43 to 0.88)	0.59 (0.42 to 0.76)	0.52 (0.29 to 0.75)	NM	NM	NM	0.01 (−0.15 to 0.17)	.85	0.09 (−0.15 to 0.33)	.44	0.08 (−0.17 to 0.32)	.52

Abbreviations: BMI, body mass index (calculated as weight in kilograms divided by height in meters squared); NM, not measured; SSBs, sugar-sweetened beverages; T1, time 1; T2, time 2; T3, time 3.

^a^ Unless otherwise indicated, data are expressed as means (95% CIs). Means are based on a mixed model with a linear link for continuous outcomes and a logistic link for dichotomous outcomes that accounts for the randomization unit of community and the community clusters within jurisdiction strata, weighted for the number of children in each community, and adjusted for child’s sex and age. The *P* values are based on a Wald test with degrees of freedom based on the number of communities.

^b^ The variables were back transformed from the regression model.

^c^ Scores range from 1 to 9, with higher scores indicating severity of sleep disturbance.

### Secondary Outcomes

There was a significant difference between the intervention and control communities from times 1 to 3 of −3.55% (95% CI, −6.17% to −0.92%; *P* = .008) for acanthosis nigricans prevalence (times 1 to 2, −6.12% [95% CI, −10.56% to −1.68%; *P* = .007]; times 2 to 3, 2.57% [95% CI, −3.61% to 8.76%; *P* = .41]). None of the behavioral variables (servings of fruit, vegetables, water, and sugar-sweetened beverages in cups per day) showed significant differences from times 1 to 3 between the intervention and control communities in similarly constructed models (eTable 2 in Supplement 2). Consumption of sugar-sweetened beverages decreased from times 1 to 3 by similar amounts in both the intervention communities and the control communities, but the difference was not significant between groups.

## Discussion

The multilevel, multicomponent CHL intervention in the USAP community-randomized trial demonstrated significant improvement in the prevalence of overweight and obesity during 4 years by an additional 8.73% from the end of the intervention (time 2), with a 12.60% decrease overall from baseline (times 1 to 3; 6 years).

 This outcome is likely due to the positive deviance strategy of supporting community initiatives that aligned with the CHL intervention template and the community-based capacity-building approach that empowered, motivated, and facilitated people and institutions to sustain change. The unifying land grant college structure in an otherwise diverse cultural and political environment provided stability. A viral-like spread was described by Swinburn et al^32^ with the Romp & Chomp study, in which a cumulative 8% decrease was found 6 years from baseline. Our 12.60% decrease at follow-up 6 years from baseline suggests a similar ongoing spread. The 2 studies were similar in age group but different in other population characteristics. Change in this young age group may have especially lasting outcomes and reach large segments of USAP populations.

The initial CHL trial prevalence difference between groups in changes in overweight and obesity of −3.88% was of similar magnitude as the difference of 3% overweight and obesity prevalence achieved in the Romp & Chomp study among preschoolers in Australia,^9^ although less than that seen in the Bright Start study.^10^ The CHL intervention, compared with the control group, resulted in a reduction in BMI *z* score of −0.04 SD units from times 1 to 2 (*P* = .39) and −0.25 SD units from times 1 to 3 (*P* = .007). The BMI *z* score effect size was similar but smaller than the difference seen in the Shape Up Somerville trial,^8^ which achieved a difference of 0.10 SD units in children 3 years of age. Our results compare favorably with those of the Shape Up Somerville trial for maintenance.^8^ Shape Up Somerville had 1 year of intervention, followed by 1 year of maintenance, compared with 2 years of intervention and maintenance assessed for BMI at 6 years in the CHL intervention.

The behavioral targets of the CHL trial did not show significant differences when tested individually during the intervention or at maintenance. Therefore, the positive obesity-related outcomes are likely a result of nudges or shifts in multiple behavioral measures,^38^ including some that were not evaluated such as other foods or the dietary pattern, stress, or gut microbiome.^39^ There may have been movement to light activity from sedentary activity that was not well captured owing to reactivity to wearing accelerometers and the inability of accelerometers to measure those changes. Owing to logistics, cost, and participant burden, the behavioral components were assessed with a smaller sample size than the primary outcomes, and loss of statistical power may play a role in those measures not showing significant change. Water intake increased and intake of sugar-sweetened beverages decreased in both the intervention and control groups, with no difference between groups at the end of intervention or at maintenance; however, the changes may have contributed to the intervention outcome. To further understand how the intervention achieved the decrease in overweight and obesity, further analysis will examine dietary patterns and apply additional systems science methods.^40^ The healthiest mix of behaviors may indeed vary in communities and jurisdictions given the varying environments in each jurisdiction. Other next steps involve new methods to describe behavioral mixes of physical activity, dietary intake, and sleep. Assessments of BMI and acanthosis nigricans raised awareness that may have helped motivate actions and that can continue to be monitored at individual, family, community, jurisdiction, and regional levels. This would not explain differences between the intervention and control groups but may serve to sustain intervention outcomes.

### Strengths and Limitations

The strengths of the CHL trial include the high-quality standardized anthropometry measurement and a common intervention template derived from a blend of community- and evidence-based approaches and actions, which provided flexibility for each community to localize and tailor the intervention to build on and strengthen local initiatives, circumstances, and aspects of Indigenous culture. The intervention approach harnessed the motivation and sense of belonging to a Pacific region–wide collective with the empowerment of local communities, leveraging both local and regional tools for sustainability. Land grant institutions provided a shared system and understanding, provided assessment and evaluation resources, and supported extension and sustainability in communities. The intervention further harnessed and was strengthened by Indigenous groups throughout the USAP region who value their culture as well as actions that benefit it.^41^ The CHL intervention built on positive aspects of the community and supported the community to expand their initiatives, strengthening existing partnerships and building on previous work. A program steering committee with a local leader facilitated collaboration along with frequent conference calls. This model has shown maintenance or sustainability of effect. The CHL trial is one of very few studies to evaluate the long-term effects of intervention sustainability 6 years after baseline.

The study limitations include possible leakage of the intervention activities to the control communities (ie, delayed optimized intervention) as well as other health-messaging campaigns throughout each jurisdiction. Although such an occurrence could compromise the outcomes of the trial, it ultimately could support comprehensive positive public health change in the region. This leakage is less likely to have occurred in temporal communities, which were not matched and tended to have other differences, such as presence on separate islands. Similarly, children could have moved from one community to another, yet this possibility was not accounted for. However, the control communities’ prevalence of overweight and obesity was higher than that of the temporal communities at time 3. Methodological limitations include a small sample size where the community is the unit of analysis in a community-randomized trial, reducing statistical power; using the number of communities as the unit for hypothesis testing likely is conservative.^42^ Although we implemented strong dietary methods, the sample size was necessarily smaller, and recording of child intake by caregivers has limitations. The acanthosis nigricans screening scale of Burke et al^33^ lacks sensitivity; however, we have shown that for population studies, the tool has utility. From a clinical perspective, because the scale is so inexpensive, it may warrant broader monitoring. The different skin complexions add complexity and error to assessment, which warrant further work to develop tools according to skin tones.

## Conclusions

The obesity- and overweight-related community changes observed in the CHL community-randomized clinical trial support the notion of multiple levels and multiple components or strategies of intervention to change and sustain a downward trajectory of overweight and obesity among children at the community level. Multiple levels, components, and strategies of intervention activities together changed the policy, system, and environmental context in which overweight, obesity, and acanthosis nigricans among children occurred. The CHL trial slowed gain in waist circumference; reduced the prevalence of overweight, obesity, and acanthosis nigricans; and showed further decreases 6 years after the initial trial among young children in communities of the USAP region.

## Notes

Supplement 1.
Trial Protocol

Supplement 2. **eTable 1.** Sample Size by Measurement Modules and by Intervention Period **eTable 2.** Differences Among Communities in the Children’s Healthy Living Trial

Supplement 3.
Data Sharing Statement
