# Supplementary material for: Maintenance Outcomes of the Children’s Healthy Living Program on Overweight, Obesity, and Acanthosis Nigricans Among Young Children in the US-Affiliated Pacific Region: A Randomized Clinical Trial
Source: JAMA Netw Open. 2022 Jun 6;5(6):e2214802. doi: 10.1001/jamanetworkopen.2022.14802 (PMC9171559; doi:10.1001/jamanetworkopen.2022.14802)
Supplement: Supplement 1. — Trial Protocol [file jamanetwopen-e2214802-s001.pdf]

The Children's Healthy Living (CHL) Program for Remote Underserved Minority Populations in the Pacific Region Randomized Clinical Trial Maintenance and Long Term Follow Up

Design and Protocol

The Children's Healthy Living (CHL) Program

The Children's Healthy Living Program for Remote Underserved Minority Populations in the Pacific Region (CHL) is a partnership among the remote Pacific jurisdictions of Alaska; American Samoa; Commonwealth of the Northern Mariana Islands (CNMI); the Freely Associated States of Micronesia (FAS) which includes the Republic of the Marshall Islands (RMI), Republic of Palau, Federated States of Micronesia (FSM); Guam; and Hawaii to study childhood obesity among Pacific children, ages 2 to 8 years. The program is sponsored by the United States Department of Agriculture (USDA), Agriculture and Food Research Initiative.

CHL Study Design

The Children's Healthy Living Program Community Randomized Trial was designed to test the intervention by comparing intervention with non-intervention communities on the prevalence of obesity in the U.S.-affiliated Pacific region collected at baseline (Time 1) and at the end of the intervention (Time 2), and then again 4 years later (Time 3), after a 6 month delayed optimized intervention in control communities and maintenance activities in intervention communities.

Objectives of the CHL Community Randomized Trial

Community Randomized Trial

The trial assessed behaviors and anthropometry of children in communities over time as indicators of whether the intervention led to change. Data has been collected at two time points – Time 1 and Time 2 (about 24 months after baseline measurement) at the end of the CHL community randomized trial, and again after 4 years of maintenance at Time 3 (about 6 years from baseline).

Objectives for the Community Randomized Trial

- Measure 2 to 8-year-old children at Time 1, Time 2 and Time 3 in selected communities to track behaviors and anthropometry that indicate healthy eating, physical activity, and BMI.
- Decrease the prevalence of young child overweight and obesity by 5%, or a reduction in 0.08 of BMI z-score, and maintain that loss over time;
- Improve and maintain the secondary outcomes related to young child overweight and obesity
    - decrease acanthosis nigricans by 5%,
    - increase sleep by 15 min/day;
    - increase moderate to vigorous physical activity by 10 min/day
    - and decrease sedentary behavior (screen time) by 10 min/day;
    - increase healthy eating (fruit and vegetable intake by 1 serving/day),
    - increase water intake by ½ cup/day;
    - decrease sweetened beverage intake by ½ cup/day,
- Develop a Pacific food, nutrition and physical activity data management and evaluation system

CHL Study Design Overview

Community / Site Selection

Communities were identified in Alaska, American Samoa, CNMI, Guam and Hawaii using the 2000 U.S. Census tract data, since 2010 data was not available at the census tract level (U.S. Census Bureau) in 2011 when sites were selected. The CHL team first selected communities based on initial eligibility criteria and then considered additional selection criteria. Based on the following criteria, communities in each of the jurisdictions were selected to participate in the intervention trial.

Community eligibility criteria:

- population size of >1000,
  - Except for FAS
- >25% of the population of indigenous/native descent
  - Except 15% in **Alaska** due to no census tract with a population of more than 1000 having more than 25% indigenous/native,

  and
- >10% of the population under age 10 years
  - (based on combining census tract data groups of < 5 years of age and 5 – 9 years of age)
  - to have sufficient population size for CHL target of 2 to 8 year olds.

Additional selection criteria:

- adequate settings for sampling and measuring children (e.g., schools);
- reasonable accessibility for the CHL team
  - (e.g., isolated communities that would require substantial travel logistics were excluded);
- community cohesiveness (Swinburn et al., 2007)

Additional selection criteria for intervention and delayed optimized (comparison) communities:

- evidence that children live and go to school in the same community
  - (i.e., not a commuter community),
- ensuring that the measured children have an opportunity to be exposed to the intervention;
- a minimal risk of contamination between matched-pair communities;
- sufficient settings for intervention (e.g., community centers, parks, churches, and stores)

A list of all eligible communities was created for each of the jurisdictions based on the above criteria. The communities were matched to form pairs based on the following factors:

- percentage in poverty and population density (both from the U.S. census),
- distance from urban centers,

- and percentage overweight/obesity, when available.

In American Samoa, CNMI, Guam and Hawaii, four communities were selected (two matched-pairs), while two communities were selected (1 matched-pair) in Alaska due to large distances between sites (see Figure 2).

In each pair, one community was randomly assigned to intervention and the other to a delayed optimized intervention (community will receive intervention at the end of the main trial). Randomization to intervention, in general, produces study groups that are comparable with respect to confounding variables (Friedman, Furberg, & DeMets, 1998). A statistician who was not part of the CHL team performed the randomization. The **delayed optimized intervention or control communities** will be called **comparison communities** in this document.

Two additional non-matched communities (third and fourth for Alaska and fifth and sixth for other jurisdictions) were selected from the eligible list of communities to serve as temporal indicators of anthropometry status. Generally, the communities selected for temporal assessment had been considered to participate as a matched pair; however, they often did not match another eligible community well or they had less community cohesiveness, which was not as important for a community providing prevalence information only. The temporal communities did not receive the intervention program as part of the CHL trial and early dissemination phase. In Guam, the two temporal communities were merged as they were found to interact as one area.

Thus, in total, four communities in Alaska, five communities in Guam, and six communities in each of the remaining four CHL intervention jurisdictions were selected for a total of twenty-seven communities across the CHL region for participation in the CHL community intervention trial: 9 matched pairs (18 sites total) and 9 temporal sites.

A cross-sectional sample of children in each of the CHL intervention communities was assessed for outcomes at Time 1 and Time 2 around 24 months from baseline, and Time 3 around 6 years from baseline.

The intervention did not explicitly target the assessed children; they served as representatives of their communities. Children who participated at multiple time points provide repeated measures and serve as an embedded longitudinal sample.

Power and sample size calculations

The process for sample size and power estimation was described in Wilken [*sic*] et al., 2013). Sample size estimates were based on the need for a sufficient number of communities and children in each of the five jurisdictions to ensure adequate statistical power to detect meaningful differences between intervention arms in overweight and related outcomes (listed previously) overall and for select outcomes within jurisdictions. The effect size, Cohen's *d,* (Cohen, 1988) was calculated based on an analysis of 2,000 simulated data sets with children clustered within community clustered within jurisdiction. The intervention effect was tested based on an F test of the interaction term of intervention group and time from a mixed model

of the outcomes, accounting for the clustering in a group-randomized trial (GRT) by adjusting the test degrees of freedom to the number of communities (Hsieh, 1988). The calculations assume a minimum *n* or sample size of 150 children with anthropometry and a minimum *n* of 100 children with accelerometry and food and activity logs in six communities in four jurisdictions and in two communities in Alaska; this assumption is conservative as the goal is a sample size of 180 children per community.

An expected correlation for communities within jurisdictions was low with an estimate of the interclass correlation coefficient (ICC) that varied between 0.02 to 0.04. We assumed a critical level of 0.05 (two-sided), a power of 80%, and a constant sample size at Time 1 and Time 2 (around 24 months). The respective effect sizes for an ICC of 0.02 and 0.04 are modest at 0.26 and 0.35 for outcomes with n=150. Using means and variances for the outcomes from previous research (de Silva-Sanigorski , 2010; Murray et al., 2004; Westerlund, Ray, & Roos, 2009), the minimum detectable differences for the two ICC values were 0.09 and 0.12 for BMI z-score, 21 and 28 minutes of television viewing, and 11 and 15 minutes of sleep. The respective effect sizes for an ICC of 0.02 and 0.04 are also modest at 0.31 and 0.42 for outcomes with n=100. Using means and variances for the outcomes from previous research (de Silva-Sanigorski , 2010; Murray et al., 2004; Ludwig, Peterson, & Gortmaker, 2001; Vorwerg, Petroff, Kiess, Bluher, 2013), the minimum detectable differences for the two ICC values were 0.50 and 0.67 servings of vegetables, 0.45 and 0.61 servings of fruits, 0.45 and 0.60 servings of water, 0.34 and 0.46 servings of SSB, and 33 and 45 minutes of PA with metabolic equivalent values (METs) > 3, based on accelerometry. The goal of 50 per community for Time 3 was set in order to detect differences compared to Time 1 or 2 with effect sizes of 0.15 and 0.18, assuming the observed ICC of 0.005.

Measures Overview

The CHL study design was to collect data on body size, functional outcomes of obesity, food intake, physical activity, lifestyle behavior which includes screen time, and demographics. These are measured through anthropometry, food and activity logs, questionnaires, and visual inspection (of the neck).

The following study outcomes were measured for children across jurisdictions using a common methodology:

Body size:

Body size measures included weight, height and waist circumference and the resultant calculations of BMI, percent overweight and obese. Trained staff in all jurisdictions **used standardized instruments, such as c**ommon scales for weight, stadiometers for height, and tape measures for waist circumference. Body size outcomes include overweight, defined as the 85th - 94th percentile for BMI (weight, kg/height, m2) and obesity, defined as greater than or equal to the 95th percentile for BMI (Centers for Disease Control and Prevention, 2009), BMI Z-score and waist circumference. During training sessions on anthropometry, inter- and intra-person reliability of each measurement,

as well as agreement to a expert measurer, were determined. We followed guidelines by Zerfas to assess agreement (1986).

Functional outcomes of obesity

Functional outcomes of obesity (Ropka, 2002) included sleep quality and duration, both as minutes per night from the accelerometer and self-reported average duration, and presence of Acanthosis nigricans as an indicator of insulin resistance/pre-diabetes.

Food intake:

We calculated nutrients and food groups of the children's diet from two days of food logs, which were completed by the parent/ caregiver, with assistance from other child caregivers. We are using these data to estimate prevalence of dietary patterns in the region. These data have been entered into PacTrac3. We used the food composition database which was developed and is maintained by the Nutrition Support Shared Resource at the UH Cancer Center. This database includes information on local foods in the Pacific region.

Physical activity:

We measured physical activity with several strategies with which we have experience – accelerometers and physical activity logs.

We developed 24-hour activity logs to measure physical activity of children in the PacDASH study, which were successfully pilot-tested for children aged 3-5 years. Parents were asked to record all activities for the child for the two days when food intake was recorded. These activity logs provided us with the type and duration of each activity of their child. Trained CHL staff assigned a metabolic equivalent (MET) that reflected the energy expenditure for the child's activity (Ridley, Ainsworth, & Olds, 2008), and a 24-hour METs could be computed.

Children were asked to wear accelerometers for six days in this study. In Year 1 of CHL, we pilot tested Actical accelerometers as a method to measure physical activity in young children to be used in the full study. Based on our successful CHL Physical Activity Pilot results, we used accelerometry at all sites (Nigg et al., 2012; Ettienne-Gittens et al., 2016, submitted). The CHL Coordinating Center (CCC) trained staff at each jurisdiction on use of the accelerometers before measurement began.

Other questionnaires:

Parents / caregiver respondents for the children completed questionnaires about demographics, lifestyle measures and culture. Lifestyle measures included food security and food expenditures (Nord, Andrews, & Carlson, 2008). In addition, parents/caregivers completed standardized questions about screen time, regarded as sedentary behavior and a lifestyle measure (Haas & Nigg, 2009).

Data Collection Visit Protocol

Measurements were taken in either a school or preschool setting (e.g., Head Start), or in a community-based setting (e.g., community recreation center or a community event) at Time 1 and at Time 2 (about 24 months), and at Time 3 (about 78 months).

Note in the temporal communities we had an abbreviated set of individual level measures, including height, weight, waist circumference and demographics.

Intervention and Comparison Communities

Parents of two to eight-year-old children were approached to learn about the study, to participate in an informed consent process and sign a consent form, to answer screening questions, and to receive instructions about completing the forms. Staff reviewed the forms for completeness as they were turned in and asked the parent to complete unanswered questions, if they were willing. All of the aforementioned may have happened at one time or over two occasions. Staff also provided training on how to complete a Food and Activity Log, using food models, etc. to demonstrate. Also, parents learned how to re-apply a wrist band and accelerometer, in the event it came off during the 6-day wearing period for the child. Parents were asked to notice if their child was still wearing the accelerometer at home and to put it back on, if the child was willing. Parents also kept a food log on their child for two days as well as an activity log for the same two days. One week after the child began to wear the accelerometer, parents sat with CHL staff to review their child's food and activity logs, and document receipt of the record. Accelerometry was not done at Time 3.

After receiving the child's assent, the anthropometry measures and the screening for acanthosis nigricans took place. Children in the intervention and comparison communities were asked to wear an accelerometer for 6 days. CHL staff asked for the child's assent and choice of wrist band before placing the accelerometer.

The protocol called for two visits by participants in intervention and comparison communities. However, for some circumstances, participants only had to attend one visit.

The circumstances for one visit were when accelerometers were not used. After a targeted number of participants wore accelerometers, they were not used in every measurement event. Also, in community events without an organization group leader who could help with follow up of retrieving accelerometers, measurement events could be held without using accelerometers.

When Food and Activity Logs (FAL) were used, but no accelerometer, sometimes participants returned their FAL by mail or to another collection site. Participants asked to return items by mail were given stamped addressed large envelopes to send their FALs back. Phone follow-up occurred as needed. In some circumstances after a targeted number of participants had already completed Food and Activity Logs, the measurement package in intervention and comparison communities did not include FAL data from participants.

Temporal Communities

Parents of 2 to 8-year-old children were approached to learn about the study, to participate in an informed consent process and sign a consent, and to receive instructions for the demographics form. Staff reviewed the form as it was turned in and asked parents about any incomplete sections. The aforementioned happened at one time or over two occasions. Their child may have been measured with the parents present or at a different time in their classroom.

Recruitment

Participant recruitment goals

In order to meet sampling goals for children between the ages of 2 – 8 years, recruitment activities involve schools and other community venues and activities. Recruitment sites consisted of Head Starts, pre-schools/day cares, kindergartens, WIC sites, community health centers and other appropriate venues (e.g., parks and community recreation centers). Recruitment efforts, led by CHL staff in each jurisdiction, involve close collaboration with community liaisons (e.g., teachers, school staff, program directors, matai, mayors) to enhance participation and retention throughout the measurement protocol. The teams in all jurisdictions tailored the recruitment strategies to work effectively with the stakeholder organizations while meeting recruitment goals of CHL.

Screening and Eligibility Criteria

Those who attended a measurement event and agreed to informed consent were asked a series of screening questions to confirm their child's eligibility. Eligibility criteria were selected for the purpose of an obesity prevention and management intervention trial. Parents of potential participants were asked to complete a screening with study staff to confirm the health status of the child.

Eligibility Criteria: The participating children will be
2-8 years old,
healthy with no known cardiovascular disease, pulmonary or metabolic disease signs and/or symptoms;
no known disease or joint problems or injuries that would be exacerbated by physical activity.
The child will be stable in the use of any prescribed medications.
The child will live in the selected community.

Exclusion Criteria:
1. Children outside the age group (under two or over eight years)
2. Known orthopedic, psychological or neurologic impairments that prevent physical activity
3. Presence or history of any metabolic or chronic health problems known to affect intermediary metabolism (e.g. untreated thyroid disease, cancer, hepatic disease, renal disease, diabetes, cardiovascular disease, hypertension)
4. Irregular use of prescription or over-the-counter medications known to affect appetite, food intake or intermediary metabolism (e.g. appetite suppressants, lithium, antidepressants, etc.

References:

1. Centers for Disease Control and Prevention. (2009). *About BMI for Children and Teens*. Retrieved from: http://www.cdc.gov/healthyweight/assessing/bmi/childrens_BMI/about_childrens_BMI.html.
2. Cohen, J. (1988). *Statistical power analysis for the behavioral sciences* (2nd edition). Hillsdale, NJ: Lawrence Erlbaum Associates.
3. de Silva-Sanigorski, A.M., Bell, A.C., Kremer, P., Nichols, M., Crellin, M., Smith, M., Sharp, S., de Groot, F., Carpenter, L., Boak, R., Robertson, N., & Swinburn, B. (2010). Reducing obesity in early childhood: results from Romp & Chomp, an Australian community-wide intervention program. *American Journal of Clinical Nutrition, 91*(4)**,** 831–840.
4. Ettienne, R., Nigg, C., Li, F., Su, Y., McGlone, K., Luick, B., Tachibana, A., Carran, C., Black, N., Mercado, J., Novotny, R., (2016). *Validation of the Actical accelerometer in multiethnic preschoolers: the Children's Healthy Living (CHL) program. Hawaii Journal of Medicine & Public Health, 75*(4), 95.
5. Friedman, L.S., Furberg, C.D., DeMets, D.L. (1998). *Fundamentals of clinical trials*. New York, NY: Spring Science-Business Media.
6. Haas, S., & Nigg, C. R. (2009). Construct validation of the stages of change with strenuous, moderate, and mild physical activity and sedentary behaviour among children. Journal of Science and Medicine in Sport, 12, 586-591.
7. Hsieh, F.Y. (1988). Sample size formulae for intervention studies with the cluster as unit of randomization. *Statistics in Medicine 7*(11)**,** 1195-1201.
8. Ludwig, D.S., Peterson, K.E., & Gortmaker, S.L. (2001). Relation between consumption of sugar-sweetened drinks and childhood obesity: a prospective, observational analysis. *Lancet*, *357*(9255), 505–508.
9. Murray, D.M., Catellier, D.J., Hannan, P.J., Treuth, M.S., Stevens, J., Schmitz, K.H., Rice, J.C., & Conway, T.L. (2004). School-level intraclass correlation for physical activity in adolescent girls. *Medicine & Science in Sports & Exercise*, *36*(5), 876–882.
10. Nigg, C., McGlone, K., Luick, B., Su, Y., Carran, C., Tachibana, A., Black, N., Novotny, R. (2012). *Physical Activity Pilot. Summary Report & Recommendations*. Honolulu, HI: Nutritional Assessment of Populations (NAP), University of Hawai'i at Manoa.
11. Nord, M., Andrews M., Carlson S. (2010). *Household food security in the United States (2008)* (Vol. 108). DIANE publishing.
12. Ridley, K., Ainsworth, B.E., Olds, T.S. (2008). Development of a compendium of energy expenditures for youth. *International Journal of Behavioral Nutrition and Physical Activity, 5*, 45. doi:10.1186/1479-5868-5-45.
13. Ropka, M.E. (2002). Symptom status and functional status outcomes: humanistic outcomes in obesity disease management. *Obesity Research. 10* Suppl 11, 42S-49S. doi: 10.1038/oby.2002.189
14. Swinburn, B., Pryor, J., McCabe, M., Carter, R.,de Courten, M., Schaaf, D., & Scragg, R. (2007). The Pacific OPIC project (obesity prevention in communities) – objectives and designs. *Pacific Health Dialog, 14*(2), 139–146.

15. U.S. Census Bureau. (*). *Census 2000 Gateway.* Retrieved from census.gov/main/www/cen2000.html
16. Vorwerg, Y., Petroff, D., Kiess, W., & Bluher, S. (2013). Physical activity in 3-6 year old children measured by SenseWear Pro(R): direct accelerometry in the course of the week and relation to weight status, media consumption, and socioeconomic factors. *PLOS ONE*, *8*, (4), e60619.
17. Westerlund, L., Ray, C., Roos, E. (2009). Associations between sleeping habits and food consumption patterns among 10-11-year-old children in Finland. *British Journal of Nutrition*, *102*(10), 1531–1537.
18. Wilken, [Wilkens],L., Novotny, R., Fialkowski, M., Boushey, C., Nigg, C., Paulino, Y., Leon Guerrero, R., Bersamin, A., Vargo, D., Kim, J., & Deenik, J. (2013). Children's Healthy Living (CHL) Program for remote underserved minority populations in the Pacific region: rationale and design of a community randomized trial to prevent early childhood obesity. *Biomed Central Public Health, 13*, 944. biomedcentral.com/1471-2458/13/944
19. Zerfas, A. J. (1986). Checking continuous measurements: Manual for anthropometry. Los Angeles: Division of Epidemiology, School of Public Health, University of California.
