# Supplementary material for: Maintenance Outcomes of the Children’s Healthy Living Program on Overweight, Obesity, and Acanthosis Nigricans Among Young Children in the US-Affiliated Pacific Region: A Randomized Clinical Trial
Source: JAMA Netw Open. 2022 Jun 6;5(6):e2214802. doi: 10.1001/jamanetworkopen.2022.14802 (PMC9171559; doi:10.1001/jamanetworkopen.2022.14802)
Supplement: Supplement 2. — eTable 1. Sample Size by Measurement Modules and by Intervention Period eTable 2. Differences Among Communities in the Children’s Healthy Living Trial [file jamanetwopen-e2214802-s002.pdf]

Supplementary Online Content

Novotny R, Yamanaka AB, Butel J, et al. Maintenance outcomes of the Children's Healthy Living Program on overweight, obesity, and acanthosis nigricans among young children in the US-affiliated Pacific region: a randomized clinical trial. *JAMA Netw Open*. 2022;5(6):e2214802. doi:10.1001/jamanetworkopen.2022.14802

eTable 1. Sample Size by Measurement Modules and by Intervention Period
eTable 2. Differences Among Communities in the Children's Healthy Living Trial

This supplementary material has been provided by the authors to give readers additional information about their work.

© 2022 Novotny R et al. *JAMA Network Open*.

eTable 1. Sample Size by Measurement Modules and by Intervention Period

	Intervention			Control			Temporal			
**Measurement Modules**	**Time 1**	**Time 2**	**Time 3**	**Time 1**	**Time 2**	**Time 3**	**Time 1**	**Time 2**	**Time 3**	**Total**
Demographic Questionnaire	1517	1342	500	1491	1295	479	1321	1405	490	9840
Context-Specific Questionnaire	1462	1277	496	1321	1261	471	0	0	483	6771
Anthropometry	1512	1309	451	1486	1268	428	1319	1369	446	9588
Acanthosis Nigricans	1509	1320	490	1479	1278	469	0	0	480	8025
Food Activity Log	922	714	101	825	684	102	0	0	105	3453
Accelerometer	893	701	N/A	731	831	N/A	0	0	N/A	3256

© 2022 Novotny R et al. *JAMA Network Open*.

eTable 2. Differences Among Communities in the Children's Healthy Living Trial[a]

Child Measure	n[b]	Intervention Communities						Control Communities						Temporal Communities					
		**T2 vs T1**	***P* value**	**T3 vs T1**	***P* value**	**T3 vs T2**	***P* value**	**T2 vs T1**	***P* value**	**T3 vs T1**	***P* value**	**T3 vs T2**	***P* value**	**T2 vs T1**	***P* value**	**T3 vs T1**	***P* value**	**T3 vs T2**	***P* value**
Anthropometry																			
Overweight and obesity prevalence, %	9473	-3.09 (-5.31, -0.87)	0.0064	-5.28 (-10.65, 0.08)	0.0535	-2.19 (-7.35, 2.96)	0.4047	0.78 (-1.63, 3.20)	0.5254	7.32 (1.86, 13.26)	0.0093	6.53 (2.72, 10.83)	0.0011	-0.89 (-4.98, 3.19)	0.6676	-1.85 (-6.63, 2.93)	0.4477	-0.95 (-4.89, 2.97)	0.633
BMI z-score	9473	-0.08 (-0.18, 0.02)	0.0935	-0.12 (-0.23, -0.02)	0.0259	-0.04 (-0.12, 0.04)	0.3127	-0.04 (-0.09, 0.01)	0.1513	0.13 (0.00, 0.26)	0.0547	0.17 (0.07, 0.27)	0.0023	-0.08 (-0.18, 0.03)	0.1401	-0.1 (-0.24, 0.04)	0.1619	-0.02 (-0.13, 0.09)	0.6925
Waist circumference, cm[b]	9308	-0.14 (-0.72, 0.45)	0.6523	0.83 (-0.06, 1.71)	0.0666	0.96 (0.20, 1.72)	0.0133	0.69 (0.40, 0.99)	<.0001	2.47 (1.53, 3.40)	<.0001	1.77 (1.03, 2.51)	<.0001	0.63 (-0.03, 1.28)	0.0601	1.19 (0.52, 1.87)	0.0005	0.57 (-0.15, 1.28)	0.119
Acanthosis nigricans prevalence, %	6544	-3.54 (-7.63, 0.55)	0.0894	-4.03 (-6.14, -1.93)	0.0002	-0.49 (-2.43, 1.45)	0.6199	2.58 (-0.02, 5.14)	0.0519	-0.49 (-2.30, 1.33)	0.5983	-3.07 (-5.34, -0.75)	0.0093	NM	NM	NM	NM	NM	NM
Behaviors																			
Sleep, h/d[b]	9724	0.44 (-0.10, 0.98)	0.1079	0.2 (-0.17, 0.56)	0.2853	-0.25 (-0.59, 0.10)	0.1564	0.56 (-0.14, 1.27)	0.1091	-0.01 (-0.24, 0.23)	0.9668	-0.57 (-1.29, 0.15)	0.1145	0.4 (-0.51, 1.31)	0.3756	0.01 (-0.41, 0.43)	0.9564	-0.39 (-1.29, 0.52)	0.3871

© 2022 Novotny R et al. *JAMA Network Open*.

Sleep disturbance[b,c]	6461	-0.36 (-0.75, 0.03)	0.0714	1.83 (0.98, 2.67)	<.0001	2.19 (1.46, 2.92)	<.0001	0.32 (-0.20, 0.85)	0.2302	1.46 (-0.18, 3.01)	0.0821	1.14 (-0.20, 2.39)	0.0981	NM	NM	NM	NM	NM	NM
Screen time, h/d[b]	6461	-0.16 (-0.46, 0.14)	0.283	0.01 (-0.48, 0.49)	0.9822	0.17 (-0.30, 0.64)	0.4819	0.31 (-0.15, 0.77)	0.185	0.1 (-0.48, 0.68)	0.7334	-0.21 (-0.80, 0.38)	0.4848	NM	NM	NM	NM	NM	NM
Dietary consumption, cups/d																			
Fruit	4966	-0.07 (-0.16, 0.01)	0.0692	0.02 (-0.10, 0.14)	0.7102	0.1 (-0.08, 0.27)	0.2538	-0.05 (-0.10, 0.01)	0.0841	-0.19 (-0.41, 0.03)	0.0897	-0.14 (-0.34, 0.06)	0.1606	NM	NM	NM	NM	NM	NM
Vegetables	4966	-0.01 (-0.05, 0.04)	0.7329	0 (-0.10, 0.10)	0.9964	0.01 (-0.08, 0.09)	0.8469	0 (-0.05, 0.05)	0.8992	-0.06 (-0.14, 0.02)	0.1304	-0.06 (-0.15, 0.04)	0.224	NM	NM	NM	NM	NM	NM
Water[b]	4966	0.01 (-0.01, 0.03)	0.3031	0.02 (-0.04, 0.08)	0.4746	0.01 (-0.04, 0.06)	0.6196	0.01 (0.00, 0.02)	0.0101	0 (-0.05, 0.06)	0.8649	-0.01 (-0.06, 0.04)	0.6909	NM	NM	NM	NM	NM	NM
SSBs	4966	-0.05 (-0.11, 0.01)	0.0955	-0.05 (-0.20, 0.11)	0.5159	0 (-0.17, 0.17)	0.9779	-0.06 (-0.22, 0.09)	0.395	-0.14 (-0.31, 0.03)	0.1078	-0.07 (-0.24, 0.09)	0.3548	NM	NM	NM	NM	NM	NM

Abbreviations: BMI, body mass index (calculated as weight in kilograms divided by height in meters squared); NM, not measured; SSBs, sugar-sweetened beverages; T1, time 1; T2, time 2; T3, time 3.

[a]Unless otherwise indicated, data are expressed as means (95% CIs). Means are based on a mixed model with a linear link for continuous outcomes and a logistic link for dichotomous outcomes that accounts for the randomization unit of community and the community clusters within jurisdiction strata, weighted for the number of children in each community, and adjusted for child's sex and age. The *P* values are based on a Wald test with degrees of freedom based on the number of communities.

[b]The variables were back transformed from the regression model.

[c]Scores range from 1 to 9, with higher scores indicating severity of sleep disturbance.

© 2022 Novotny R et al. *JAMA Network Open*.

eTable 2. Differences Among Communities in The Children's Healthy Living Trial[a] (cont.)

Child Measure	n[b]	Intervention vs Temporal Communities					
		T2 vs T1	*P* value	T3 vs T1	*P* value	T3 vs T2	*P* value
Anthropometry							
Overweight and obesity prevalence, %	9473	-2.2 (-6.72, 2.32)	0.3411	-3.43 (-10.36, 3.50)	0.3316	-1.29 (-7.74, 5.27)	0.7101
BMI z-score	9473	-0.01 (-0.15, 0.14)	0.9272	-0.02 (-0.19, 0.15)	0.7697	-0.02 (-0.17, 0.13)	0.8055
		-0.76 (-1.75, 0.23)	0.1317	-0.37 (-1.42, 0.68)	0.4919	0.39 (-0.67, 1.46)	0.468
Waist circumference, cm[b]	9308	NM	NM	NM	NM	NM	NM
Acanthosis nigricans prevalence, %	6544	NM	NM	NM	NM	NM	NM
Behaviors							
Sleep, h/d[b]	9724	0.04 (-1.13, 1.21)	0.9405	0.18 (-0.25, 0.65)	0.5165	0.32 (-0.93, 1.21)	0.7866
Sleep disturbance[b,c]	6461	NM	NM	NM	NM	NM	NM
Screen time, h/d[b]	6461	NM	NM	NM	NM	NM	NM
Dietary consumption, cups/d							
Fruit	4966	NM	NM	NM	NM	NM	NM

© 2022 Novotny R et al. *JAMA Network Open*.

Vegetables	4966	NM	NM	NM	NM	NM	NM
Water[b]	4966	NM	NM	NM	NM	NM	NM
SSBs	4966	NM	NM	NM	NM	NM	NM

Abbreviations: BMI, body mass index (calculated as weight in kilograms divided by height in meters squared); NM, not measured; SSBs, sugar-sweetened beverages; T1, time 1; T2, time 2; T3, time 3.

[a]Unless otherwise indicated, data are expressed as means (95% CIs). Means are based on a mixed model with a linear link for continuous outcomes and a logistic link for dichotomous outcomes that accounts for the randomization unit of community and the community clusters within jurisdiction strata, weighted for the number of children in each community, and adjusted for child's sex and age. The *P* values are based on a Wald test with degrees of freedom based on the number of communities.

[b]The variables were back transformed from the regression model.

[c]Scores range from 1 to 9, with higher scores indicating severity of sleep disturbance.

© 2022 Novotny R et al. *JAMA Network Open*.
