# Supplementary material for: Maintenance Outcomes of the Children’s Healthy Living Program on Overweight, Obesity, and Acanthosis Nigricans Among Young Children in the US-Affiliated Pacific Region: A Randomized Clinical Trial
Source: JAMA Netw Open. 2022 Jun 6;5(6):e2214802. doi: 10.1001/jamanetworkopen.2022.14802 (PMC9171559; doi:10.1001/jamanetworkopen.2022.14802)
Supplement: Supplement 3. — Data Sharing Statement [file jamanetwopen-e2214802-s003.pdf]

Data Sharing Statement

Novotny. Maintenance Outcomes of the Children's Healthy Living Program on Overweight, Obesity, and Acanthosis Nigricans Among Young Children in the US-Affiliated Pacific Region. *JAMA Netw Open*. Published June 06, 2022. doi:10.1001/jamanetworkopen.2022.14802

Data
Data available: Yes
Data types: Deidentified participant data, Data dictionary
How to access data: https://www.chl-pacific.org/chl-data/data-requests/
When available: With publication

Supporting Documents
Document types: None

Additional Information
Who can access the data: investigators who are able to fulfill the requirements of our data sharing agreement
Types of analyses: A research question that is approved by the program steering committee.
Mechanisms of data availability: ethics training, data sharing agreement, approved proposal
